# Optimized method to harvest both kidneys from one donor rat for transplantation

**DOI:** 10.1186/s12893-018-0400-9

**Published:** 2018-08-29

**Authors:** Chun-hua Ju, Ling-na Xue, Hong-jia Cheng, Zhong-da Jin

**Affiliations:** 1 Department of Kidney Center, The Second Affiliated Hospital of Guangzhou University of Chinese Medicine, Guangdong Provincial Hospital of Traditional Chinese Medicine, 111 Dade Road, Yuexiu District, Guangzhou, 510120 Guangdong Province China; 2 The Second Clinical Collage of Guangzhou University of Chinese Medicine, Guangzhou, 510405 Guangdong Province China

**Keywords:** Kidney transplantation, Microsurgery, Model, Rat, Warm ischemia

## Abstract

**Background:**

Rat renal transplantation is an essential experimental model for studies of transplantation immunobiology. Harvesting both kidneys from one donor rat for transplantation is widely used to reduce the number of experimental animals. Using the conventional method, both kidneys of the donor rat are harvested simultaneously, which leads to the prolonged warm ischemic times during transplantation of the second donor kidney. Prolonged warm ischemia time is the main risk factor for delayed graft function.

**Methods:**

Two different approaches are compared. Method 1, conventional method: both kidneys of the donor rat are harvested simultaneously and then transplanted into two recipients. During transplantation, the first and second donor kidneys were regarded as Group 1 and 2, respectively. Method 2, step-by-step method: after left nephrectomy, the donor rat survives, and we perform left renal transplantation (Group 3). Then, the right kidney of the surviving donor rat is incised and transplanted into the left side of the second recipient (Group 4).

**Results:**

The success rates were 86.7, 93.3, 93.3 and 86.7% in groups 1, 2, 3 and 4, respectively. The warm ischemia times increased significantly in group 2 compared with the other 3 groups (*p* < 0.05) but differed non-significantly between groups 3 and 4 (*p* > 0.05). Serum creatinine levels, blood urea nitrogen and 24-h urine protein level obviously increased after kidney transplantation in group 2 compared with other groups (*p* < 0.05).

**Conclusions:**

We developed an optimized method for reducing warm ischemia time, thereby minimizing delayed graft function.

## Background

In recent years, bilateral donor nephrectomy in rat kidney transplantation has been commonly used because it can save time and laboratory animals. However, harvesting two kidneys from one donor simultaneously and then transplanting the kidneys cannot guarantee identical warm ischemia times for the two donor kidneys in the case of a single-person operation, and prolonged warm ischemia time is the main risk factor for delayed graft function [1]. Wagner reported that the surgical difficulty of rat renal transplantation was the second level [2], requiring the aid of microsurgical equipment and greater than 6 months of microsurgical training. This article seeks to solve this problem by employing an optimized method to harvest two kidneys from one donor step-by-step, thereby ensuring that the warm ischemia time of the first donor graft is almost equal to the second graft in an individual. The process is reported as follows.

## Methods

### Animals and experimental design

This study were performed at the Laboratory Animal Center of Guangzhou University of Chinese Medicine (Guangzhou, China) in accordance with the Care and Use of Laboratory Animals (NIH publication 86–23, revised 1985). Adult male inbred Brown Norway (BN) and Lewis rats (weight 200–250 g) were used as donors and recipients, respectively. The rats were supplied by Vital River Laboratory Animal Technology Co. Ltd. (Beijing, China). The animals were fasted 10 h before operation but had free access to water.

Method 1, conventional method: Both kidneys were harvested from BN rats (*n* = 15) and transplanted into 30 Lewis rats. Both kidneys of the donor rat are harvested simultaneously and then transplanted into two recipients. During transplantation, the first and second donor kidneys were regarded as Groups 1 and 2, respectively. Method 2, step-by-step method: after left nephrectomy, the donor rat (*n* = 15) survives, and we performed left renal transplantation (Group 3, *n* = 15). Then, the right kidney of the surviving donor rat is incised and transplanted to the left side of the second recipient (Group 4, *n* = 15). In all groups, renal artery (RA) revascularization was performed in an end-in-end fashion via modified sleeve anastomosis [3, 4]. Renal vein (RV) anastomosis was performed end-to-end using a modified stenting technique [4, 5]. The ureter was anastomosed end-to-end using 6 interrupted equally spaced sutures.

### The first stage: Left donor nephrectomy

**Fig. 1:**
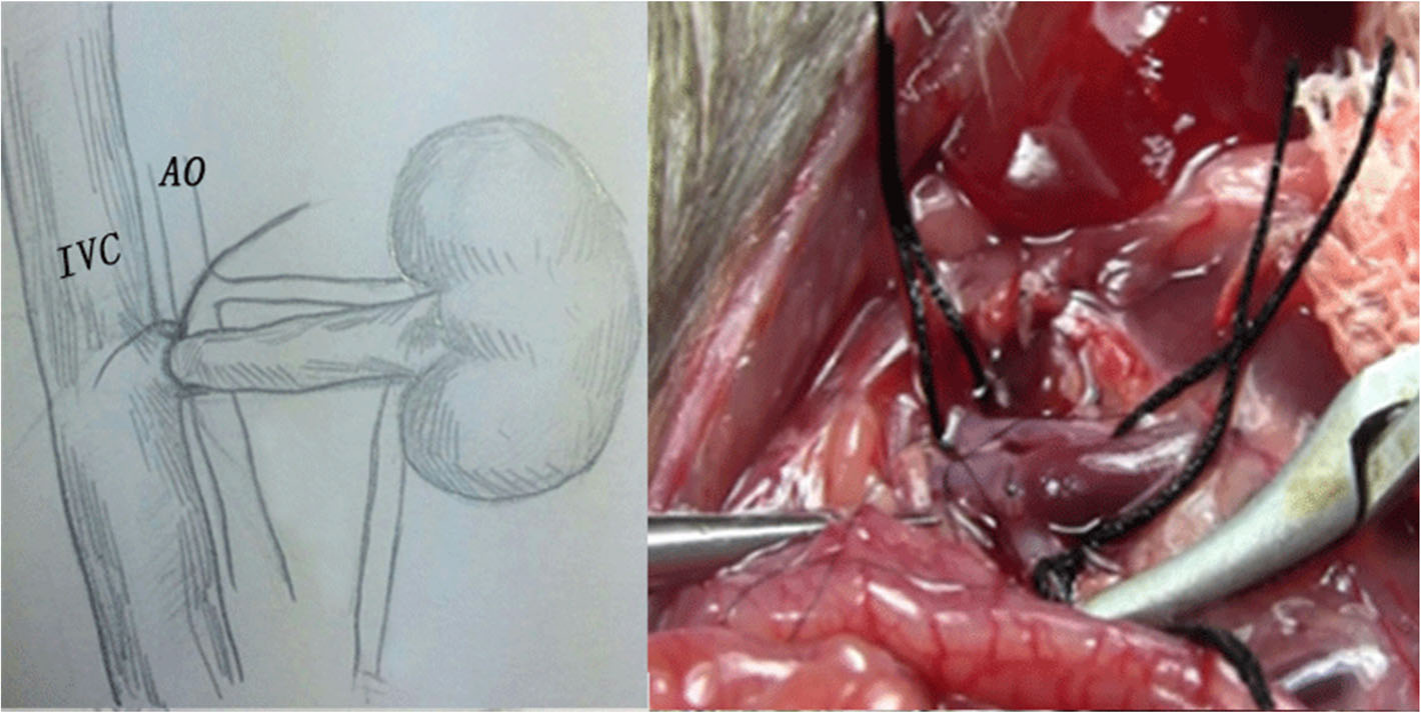
The donor left RA and RV were separated entirely before perfuse the left renal from the AO, a suture line was placed in the left RV just close to the IVC, tied and cut

**Fig. 2:**
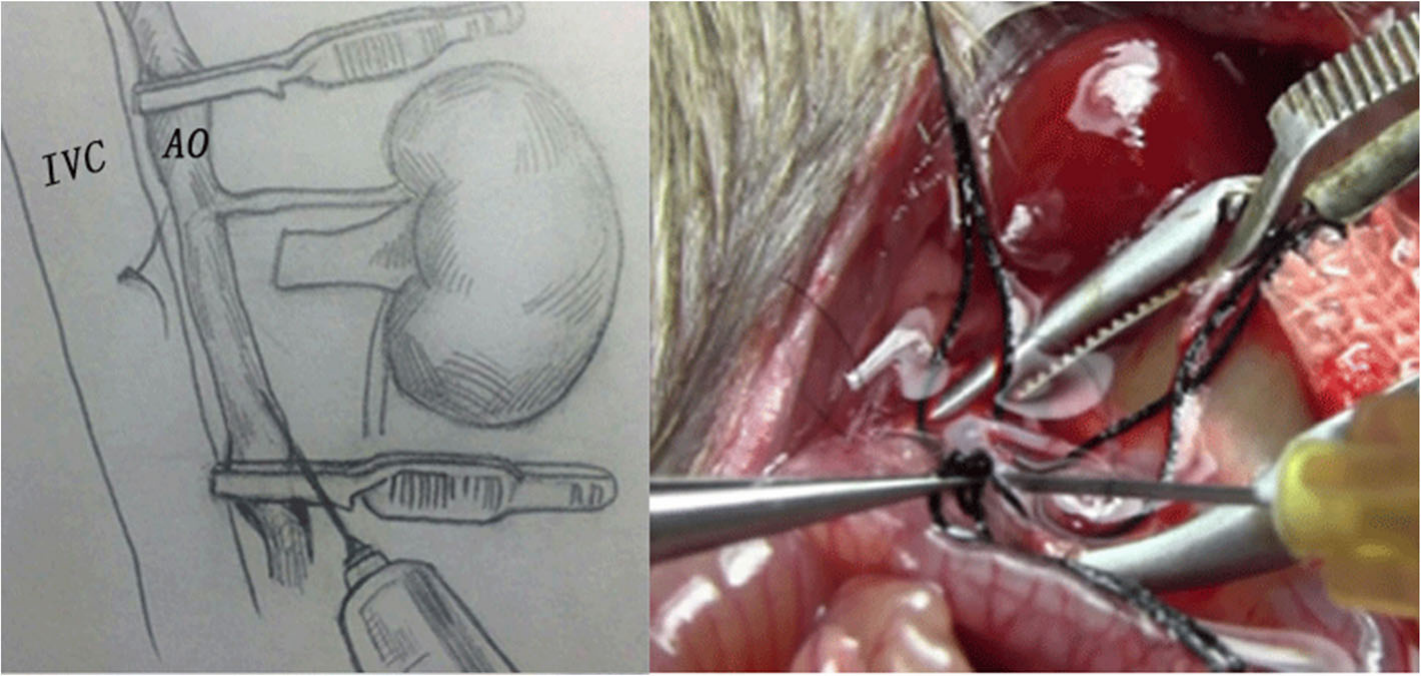
Four vascular clips were used to block the AO and IVC at the distal point;The graft was then perfused slowly from the AO until the left kidney became uniformly pale and there was a clear perfusate from the RV

**Fig. 3:**
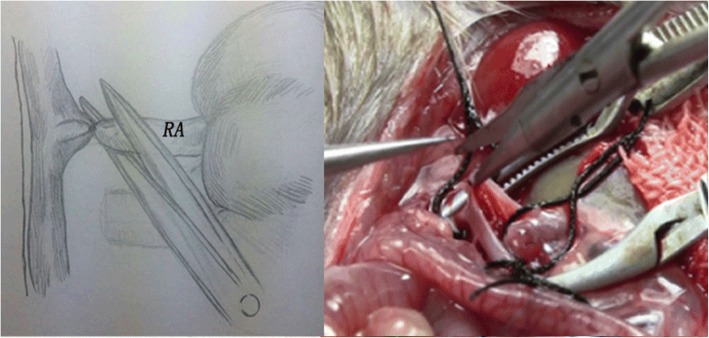
The RA root proximal to the AO was cut and the graft was prepared to removed

The operation was performed by an individual, under a 10× binocular operating microscope. Anesthesia was induced and maintained with chloral hydrate (50 μg/μl, 6 μl/g) via intraperitoneal injections. A long midline abdominal incision was generated, and two retractors were placed to expose the viscera. The bowel was retraced to the right. We can observe the left kidney, abdominal aorta (AO), and inferior vena cava (IVC), and then separated the branches of the vessels, such as the left adrenal artery and vein and testicular vein, ligated, and cut. The left RA and RV were separated at the bifurcation of the AO and IVC, respectively. At this time, the AO and IVC were completely divided. We mobilized the adipose tissue by sharp dissection at about the upper part of the left ureter, preserving adherent ureteral connective tissue and fat to ensure blood supply. The ureter was cut approximately 10 mm to the renal hilum. The distal end of the ureter was ligated. At this moment, the whole left kidney, including the RA, RV, and ureter, was dissected free. Two vascular clips were used to block the AO and IVC together at the lower and upper levels of the left renal vessels. The left RV root proximal to the IVC was tied and transected (Fig. 1). A needle was inserted in the middle of the blocked AO (Fig. 2). After the graft was perfused with 10 ml of heparin physiological saline (25 U/ml, 4 °C), the RA root proximal to the AO was tied and cut (Fig. 3). The ureter was then cut, and the left graft was placed in ice physiological saline in preparation for transplantation. A cross suture was made on the side of needle insertion into the AO. Vascular clips were loosed. After left kidney removal, the donor rat was slowly injected with 3 ml physiological saline via the penile vein. The abdominal viscera were replaced in their original position, and the abdominal wound was sutured continuously in layers. The donor rat survived the operation and was prepared for right donor nephrectomy.

### Recipient operation

The steps for preparation of the recipient rat are basically the same as the donor. After separation of the left RA and RV, the RA and RV were clamped by two vessel clips as close to the AO and IVC as possible. The surrounding tissues of the ureter were mobilized properly, and then the ureter was cut at about the lower pole of the renal. At last, the left kidney under the capsule was excised. RA revascularization was performed in an end-in-end fashion via modified sleeve anastomosis [3, 4]. RV anastomosis was performed using a modified stenting technique [4, 5].

After the RA and RV anastomoses were complete, the graft was reperfused. As urine passed from the ureterostoma, we began to stitch the ureter. A 10–0 suture line was used to anastomose the ureter end-to-end with 6 interrupted sutures. Finally, in order to hold the graft, two stitches were placed in the upper and lower poles of the donor kidney, respectively. The right renal of the recipient was removed and the first step of left donor orthotopic transplantation was complete.

### The second step: right donor nephrectomy

**Fig. 4:**
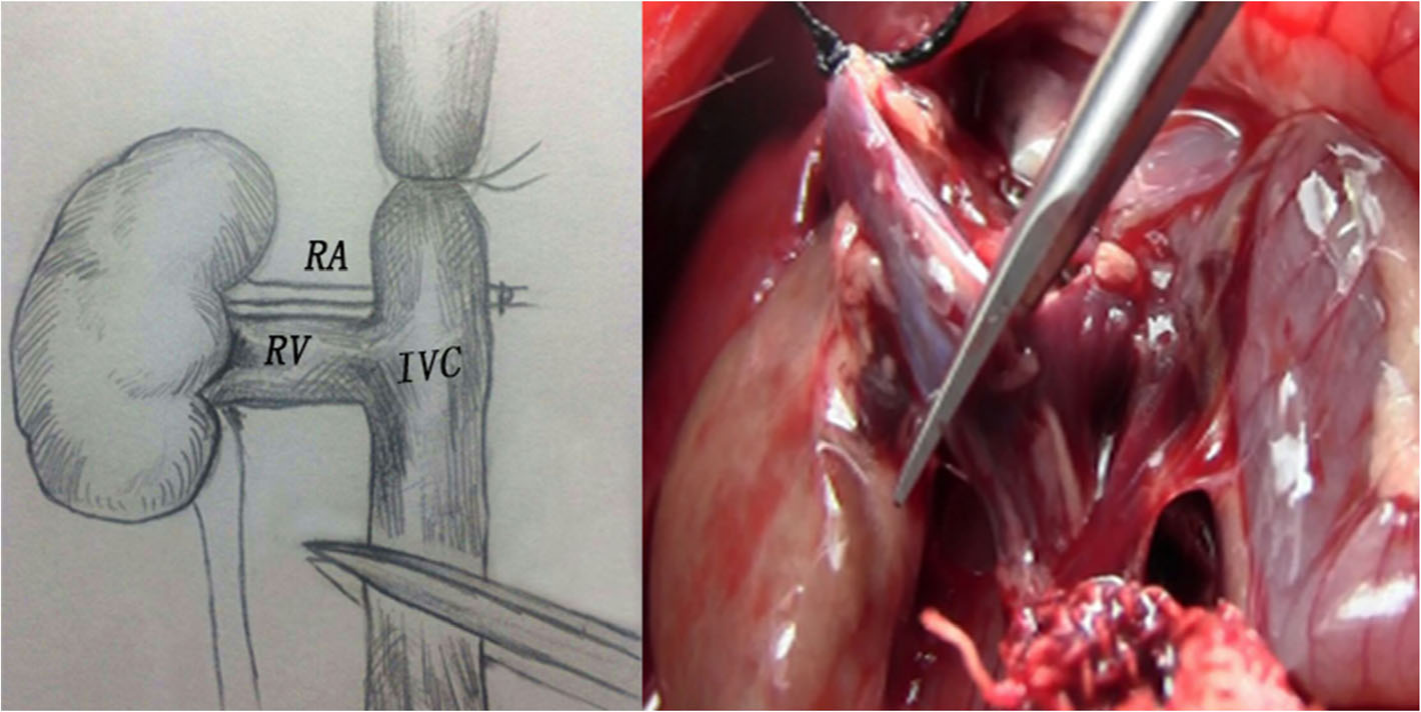
The right graft was perfused slowly from the AO and the IVC above the RV was tie and got ready to cut below the RV

The donor rat subject to left nephrectomy was prepared as described in the first step. After entering abdominal cavity, the bowel was retraced to the left and the right kidney, and AO and IVC were exposed. The AO, IVC, right RA, RV and ureter were separated as described for the left side. After separating the right renal, four vascular clamps were used to block the AO and IVC at both upper and lower ends. The IVC were cut approximately 2 mm below the right RV to anastomose with the recipient RV. Similar to left graft perfusion, the right renal was perfused in situ, and a clear perfusate was obtained from the RV with a segment of the IVC. Thereafter, the right RA was transected close to the AO. The IVC above the right RV was ligated using a 5–0 suture line and cut at the distal end (Fig. 4). The right kidney was immediately transferred to physiological saline at 4 °C.

### The right kidney transplantation

The right donor kidney was rotated 180 degrees around its longitudinal axis. The vessel and ureter anastomosis processes were the same as described for the left renal orthotopic transplantation.

### Postoperative condition

Postoperatively, the rats were placed under a heating mat and recovered from the anaesthesia. Blood samples were obtained from the caudal vein. Urine samples were obtained at 1, 5 and 7 days postoperatively for assessment of serum creatinine levels, blood urea nitrogen and 24-h urine protein levels. After the experiment, all animals were euthanized by cervical dislocation.

### Statistical analysis

Statistical analysis was performed in SPSS 20.0 (Statistical Product and Service Solutions software). Data are reported as the mean values ± standard deviations. Student’s t-test was used to compare the quantitative parametric data, and the χ^2^ test was used to compare qualitative data between the experimental and control groups. *p* < 0.05 was considered statistically significant.

## Results

In Table 1, serum creatinine, blood urea nitrogen and 24-h urine protein levels increased significantly at 1, 5 and 7 days after kidney transplantation among all groups and obviously increased in group 2 compared with other groups (*p* < 0.05). In Table 2, the warm ischemia times increased significantly in group 2 compared with the other 3 groups (*p* < 0.05) but exhibited no significant difference between groups 3 and 4 (*p* > 0.05). The warm ischemia times increased significantly in group 2 compared with the other 3 groups (*p* < 0.05) but exhibited no significant difference between groups 3 and 4 (*p* > 0.05). The success rates were 86.7, 93.3, 93.3 and 86.7% in groups 1, 2, 3 and 4, respectively. No differences were noted among the four groups (*p* > 0.05). The main complications after operations were venous thrombosis and ureteral leakage in all animals; however, complications rarely occurred in all groups.

**Table 1:** Comparison of Serum Creatinine Levels (SCr, μmol/L), Blood Urea Nitrogen (BUN, mmol/L) and 24-h urine protein Quantity (24 h UPQ, mg/24 h) Among 4 Groups at Different Time Periods After Transplantation

Group	Postoperative (d)											
	Preoperative			1			5			7		
	Scr	BUN	24 h UPQ	Scr	BUN	24 h UPQ	Scr	BUN	24 h UPQ	Scr	BUN	24 h UPQ
1(*n* = 15)	67.5 ± 4.8	8.1 ± 1.5	5.2 ± 2.3	80.3 ± 5.4	12.8 ± 1.2	6.2 ± 2.6	150.3 ± 16.1	15.4 ± 1.1	10.8 ± 5.2	310.9 ± 21.3	18.7 ± 1.6	15.3 ± 3.7
2(*n* = 15)	62.6 ± 6.1	8.6 ± 2.0	5.6 ± 1.6	95.2 ± 17.3 †	15.0 ± 2.3 †	8.9 ± 0.9 †	198.4 ± 12.3 †	19.5 ± 1.5 †	16.7 ± 3.3 †	330.9 ± 21.0 †	25.2 ± 1.8 †	19.0 ± 2.5 †
3(*n* = 15)	64.4 ± 5.2	7.8 ± 1.9	6.4 ± 2.0	81.5 ± 6.8	12.1 ± 1.5	7.0 ± 2.4	160.2 ± 15.7	15.9 ± 2.1	10.6 ± 5.9	305.3 ± 22.1	20.3 ± 2.7	15.9 ± 3.2
4(*n* = 15)	66.8 ± 5.9	8.4 ± 1.8	6.5 ± 1.7	82.6 ± 3.3	13.0 ± 1.3	6.9 ± 1.2	162.4 ± 18.3	16.1 ± 1.0	9.9 ± 4.7	300.5 ± 26.0	19.2 ± 2.2	16.0 ± 2.9

† Compared with other groups at each time point *P*<0.05

**Table 2:** Operation Time and Complications of 4 Groups

Parameters	Group 1	Group 2	Group 3	Group 4	*p*-value
Warm ischemia time, min	24.7 ± 2.3	95.3 ± 3.6 †	26.0 ± 1.8 ‡	25.3 ± 1.4 ‡	–
Venous thrombosis, %	6.7(1/15)	0(0/15)	6.7(1/15)	6.7(1/15)	>0.05
Ureteral leakage, %	6.7(1/15)	6.7(1/15)	0(0/15)	6.7(1/15)	>0.05
Recipient survival rates after 3 days, %	86.7(13/15)	93.3(14/15)	93.3(14/15)	86.7(13/15)	>0.05

† Compared with other groups *P*<0.05

‡ There are insignificant differences between the data compared. (*P*>0.05)

## Discussion

In recent years, using both kidneys from one donor rat for renal transplantation has become a commonly applied method to save animals. Yin et al. [6] employed bilateral donor nephrectomy. The donor IVC and AO were end-to-side anastomosed with the recipient IVC and AO, respectively. Feng et al. [7] reported donor RA end-to-side anastomosed with the recipient AO. An end-in-end technique was used for the RA, and the bladder-patch method was employed for urinary tract reconstruction. However, all the techniques described above blocked the bloodstream of the AO and IVC to various degrees. Thus, the hemodynamics were significantly altered after ischemia. Blood clots easily form during reperfusion in the recipient rat. The bladder-patch technique affects the rat’s recovery after surgery due to large operative wounds. Without blocking the AO and IVC, vessel and urinary tract reconstruction via end-to-end anastomosis has been widely adopted in experimental research given its high success rate and low complication rate [8]. Grau et al. [9] harvested two kidneys from one donor and completed rat kidney transplantation using an end-to-end anastomosis technique for the RA, RV and ureter. However, vessel anastomosis requires a high level of technical expertise, especially for RV anastomosis given that the vessel wall is weak and difficult to distinguish. Thus, the technique is difficult to perform in a single-person operation. When harvesting two kidneys from one donor rat for transplantation, both kidneys must be cut and perfused simultaneously. If the recipient operation was not manipulated simultaneously by two individuals, the warm ischemia time would exhibit significant variation between the two grafts, and the interventional factors of the experimental study will increase. Prolonged warm ischemia time is the main risk factor for delayed graft function [1].

Rat kidney transplantation requires advanced microsurgery techniques and skills due to vessel and ureter anastomosis, and the techniques are often performed by one person. Our study explored a new technique to harvest two kidneys from one donor in a step-by-step fashion for a single-person operation to ensure that the warm ischemia time of the left donor graft is almost equal to that of the right graft and protect renal function to the maximal extent. The donor rat survives after left nephrectomy. After the left kidney transplantation was completed, we incise the right graft of the surviving rat. Our new step-by-step technique was first described at home and abroad. The results reveal wide variation in the warm ischemia time between groups 1 and 2 when the conventional method is employed. In the step-by-step method, the warm ischemia time of the left and right donor graft did not differ significantly between groups 3 and 4 (*p* > 0.05). Moreover, the extension of the warm ischemia time causes more rapid disease progression in acute renal allograft rejection. In addition, serum creatinine, blood urea nitrogen and 24-h urine protein levels increased significantly in group 2 compared with other groups (*p* < 0.05). This finding indicates that the warm ischemia time is closely related to the recovery of renal function after transplantation. This finding is explained as follows: the kidney is one of the organs with the largest blood supply in the entire body and is sensitive to ischemia. If the warm ischemia time is longer, the reperfusion injury is more serious. Our study results are consistent with previous results [1].

## Conclusions

Under individual circumstances, our step-by-step methods ensure that the warm ischemia time of the left donor graft is approximately equal to the right graft, thus minimizing delayed graft function and preserving animals. Our technique exhibits a high success rate and low complication rate.

## Abbreviations

AO: abdominal aorta
BN: Brown Norway
IVC: Inferior vena cava
RA: Renal artery
RV: Renal vein
